# Habitat characteristics or protected area size: What is more important for the composition and diversity of mammals in nonprotected areas?

**DOI:** 10.1002/ece3.7540

**Published:** 2021-05-01

**Authors:** Wenbo Li, Peipei Yang, Bowen Li, Chao Liu, Lixing Sun, Jinhua Li

**Affiliations:** ^1^ School of Resources and Environmental Engineering Anhui University Hefei China; ^2^ International Collaborative Research Center for Huangshan Biodiversity and Tibetan Macaque Behavioral Ecology Anhui University Hefei China; ^3^ Department of Biological Sciences Central Washington University Ellensburg WA USA; ^4^ School of Life Sciences Hefei Normal University Hefei China

**Keywords:** camera traps, conservation management, habitat characteristics, mammal diversity, protected areas size

## Abstract

The margins of protected areas are usually considered to have greater forest degradation, and given that most mammals live outside protected areas, researchers and conservation practitioners are increasingly recognizing that nonprotected areas must be incorporated into conservation strategy. However, the strategy used to manage these areas still involves increasing the size of protected areas, while not considering the habitat characteristics and requirements of the species. In this study, during a 3‐year period, camera trap and habitat characteristic surveys were used to estimate composition, diversity, and habitat characteristics of mammals to determine habitat characteristics or increase the size of protected areas what should be considered first for mammals’ conservation in a nonprotected area near the Huangshan Mountains in Anhui Province, China. From June 2017 to October 2019, 18 species of mammals were recorded, more than in any other protected area nearby. The linear model analysis results showed that habitat characteristics of mammals were different and showed a significant correlation with their relative abundance. Most species were related to vegetation characteristics, except primates (*Macaca thibetana*), and rodents (*Leopoldamys edwardsi*). Therefore, to establish conservation policies for nonprotected areas, habitat characteristics should be of prime concern, followed by increasing the size of protected areas to provide effective refuge areas for species conservation.

## Graphical abstract

Very few studies (and that too lacking systematic and serious research efforts) have been done in areas surrounding protected areas and natural reserves. There are many knowledge gaps in the understanding of variations in communities or assemblages of mammals in unprotected subtropical forests near protected area boundaries. This study investigated this gap in knowledge.

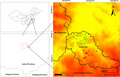

## 1 INTRODUCTION

The most effective strategy for species conservation is to protect all their habitats. However, it is impossible to do in this way, the common practice is building nature reserves or protected areas to conserve original forest habitat characteristics for endangered species, achieving the goal of conserving the species, but the quality of enforcement in this protected areas is highly variable (Astudillo‐Scalia, & de Albuquerque, 2020; Brum et al., 2017; Burns et al., 2003). A large number of protected areas still face problems such as illegal hunting (Duporge et al., 2020), wildfires (Camargo et al., 2018), and deforestation (Mekonen, 2020); thus, some forest's species diversity became very low or even extinct and finally formed the “empty forest” phenomenon (Redford, 1992). This empty forest is increasingly common in many protected areas, directly related to the effectiveness of new protected areas.

However, with the implementation of the global biodiversity conservation plan, there are still large biodiverse areas that are not classified as protected (Burns et al., 2003; McShea et al., 2009), even though they have been shown to have high species diversity, which may provide germplasm resources or gene flow for threatened species in protected areas, maintaining biodiversity (Yahner, 1988), and study on the genetic diversity of the giant anteater (*Myrmecophaga tridactyla*) has identified the migration between the different populations influenced by fragmented habitats (Sartori et al., 2021). Under adequate government protection and the improvement of public awareness in conservation, the habitat characteristics of unprotected areas/biological corridors are slowly returning to the level of protected areas. The "empty forests" are also being resettled by more species from protected areas. However, habitat characteristics in these areas, such as understory vegetation, medium‐ and small‐sized animals, it is vulnerable to redestruction by logging, illegal hunting, natural disasters, etc. require immediate conservation and management intervention (Bai et al., 2020; Dorji et al., 2019). Also, the restoration in these areas can only satisfy certain species with specific habitat characteristics. Therefore, such factors should be considered in the planned establishment of new protected areas/corridors or the improvement of existing protected areas.

Animals have potential for habitat selection; studies have shown that although they can spread into unoccupied areas, most animals cannot fully monopolize their potential habitats (Bai et al., 2020; Sukma et al., 2019; Wong et al., 2018). This means that they only select to live in some fixed habitat, which can meet their requirements of habitat characteristics (Huang et al., 2020). In addition, the difference in habitat characteristics (such as geography, food, and the distribution of other species) has an impact on animal distribution (Huang et al., 2020). Therefore, the habitat characteristics of animals should be considered when undertaking animal protection strategies. Many protected areas have been created without consideration of habitat characteristics, which has eventually resulted in lower conservation effectiveness of nature reserves, leading to problems such as animal population decline (Craigie et al., 2010; Kolahi et al., 2013; Scott et al., 2001; Xu et al., 2014).

Mammals are important components of forests; however, only a few studies are available that provide information on the species composition and diversity of mammals especially in nonprotected areas for the lower mammals diversity and low density of endangered species (Bogoni et al., 2016; Hagger et al., 2013; Wang, 1990). However, the situation conversed after the mammals' diversity of “empty forest” gradually recovered. For instance, conversion of forests into secondary forests does not always result in mammal species decline, as some species thrive—for example, Squirrels (*Callosciurus erythraeus*) and Wild boar (*Sus scrofa*) (Meijaard & Sheil, 2007)—depending on forest mosaic, tree species composition, structure, type, age, and the number of predators in the forests. Forest structure changes make the understory vegetation more open, and the increase of herbaceous layer coverage is also beneficial to ungulates such as Reeve's muntjac (*Muntiacus reevesi*). Otherwise, such habitats can also cause specialists and human‐sensitive species to decline, such as felid species guilds (Cheyne et al., 2016; Chiang et al., 2014) and Wildebeest (*Connochaetes taurinus*) (Craigie et al., 2010; Thirgood et al., 2004). These species may travel to nonprotected areas at certain times for migration or to maintain large home ranges. While outside of protected areas these populations may be exposed to higher levels of threats such as hunting, these population changes may not be solely reliant on conditions inside protected areas but also conditions outside. Besides, mammals may affect the structure and composition of forests by feeding on seeds and spreading them, makings the ecology restoration in secondary forests more quickly, eventually attracting more animal resettlement (Andresen et al., 2018; Fedriani & Delibes, 2009). Similarly, activities, such as trampling, wallowing, and digging, by wild boar may physically alter the substrate and the vegetation structure (Barrios‐Garcia & Ballari, 2012). The existence of some predators (e.g., Clouded leopard (*Neofelis nebulosa*) and Leopard cat (*Prionailurus bengalensis*)) can also control the destruction of animal ecosystems caused by excessive growth of other small mammals (Kolchin, 2018; Watanabe & Izawa, 2005). Therefore, it is necessary to understand the diversity, composition, and habitat characteristics of mammals in nonprotected areas.

The subtropical forest in Mt. Huangshan is among the most diverse in the world and an important member of the 32 inland biodiversity protection areas in China (Huangshan–Huaiyu mountain area). Several studies have focused on mammals and bird communities in this forest, and all have been conducted in protected areas. None of the studies have been studied in nonprotected areas. In addition, most of these studies are tentative, lacking systematic and regular research efforts, and it was just a basic survey of species (Fang, 2017; Liu et al., 2017; Wang et al., 2015). Mammals constitute a key component of tropical and subtropical forest ecosystems (Wang, 1990). However, there are many knowledge gaps in the understanding of variations in communities or assemblages in unprotected subtropical forests stationed as protected area boundaries.

Mammals display a wide array of body size, behavior (e.g., arboreal, terrestrial, diurnal, and nocturnal), and home range size, which makes it challenging to conduct standardized surveys on subtropical forest mammals. Several methods of sampling mammalian fauna have been tried and tested with limited success. Evidently, no single approach or technique has proven suitable for conclusively surveying the entire mammalian fauna (O’Connell et al., 2011). Recently, camera traps have become an important tool for terrestrial species surveys, in particular for mammal surveying (Andresen et al., 2018; Blake & Loiselle, 2018; McShea et al., 2009; Srivastava & Kumar, 2018; Widness & Aronsen, 2018). The method has also been used to either research or study such as the effects of human disturbance and environmental change on mammals (Vanthomme et al., 2013), the conservation of species that are rare and endangered, for animal monitoring in human landscapes, and in behavior studies of nonhuman primates (Pebsworth & LaFleur, 2014; Rabinowitz & Nottingham, 1989; Saito & Koike, 2013), to document the use of specific habitats by animals (Fiderer et al., 2019; Granados et al., 2016).

Mammalian species composition and diversity show obvious spatio‐temporal dynamic changes and are directly related to the spatial scale of studies. Heterogeneous habitats influence species distribution and abundance patterns, including temporal variation in environmental conditions (Bhattarai & Kindlmann, 2011; Blake & Loiselle, 2018; Saito & Koike, 2013; Vanthomme et al., 2013). Several studies have employed the use of camera traps to survey mammalian communities cover vast land areas but may not reveal patterns of activity concerning smaller‐scale differences in habitat (Mochizuki & Murakami, 2013). Temporal activity and local‐scale distribution patterns may reflect small‐scale variation in habitat environments (Meijaard & Sheil, 2007; Widness & Aronsen, 2018). Here, we study small‐scale patterns of mammal activity using camera traps in small nonprotected areas to ensure reliable sampling effort. We aim to answer the question of whether concentrate on habitat characteristics or increase the size of protected areas when considering the biodiversity conservation strategies in new nonprotected areas. We hypothesized that (a) nonprotected areas have more mammal species than protected areas because of the edge effect (Cheyne et al., 2016; Yahner, 1988) and (b) nonprotected areas have more species associated with habitat characteristics (Bhattarai & Kindlmann, 2011; Blake & Loiselle, 2018). We predicted that habitat characteristics should be considered first and increase the size of protected areas second in the nonprotected area as these areas are the last refuge for mammals.

## 2 METHODS

### 2.1 Study sites

**FIGURE 1:**
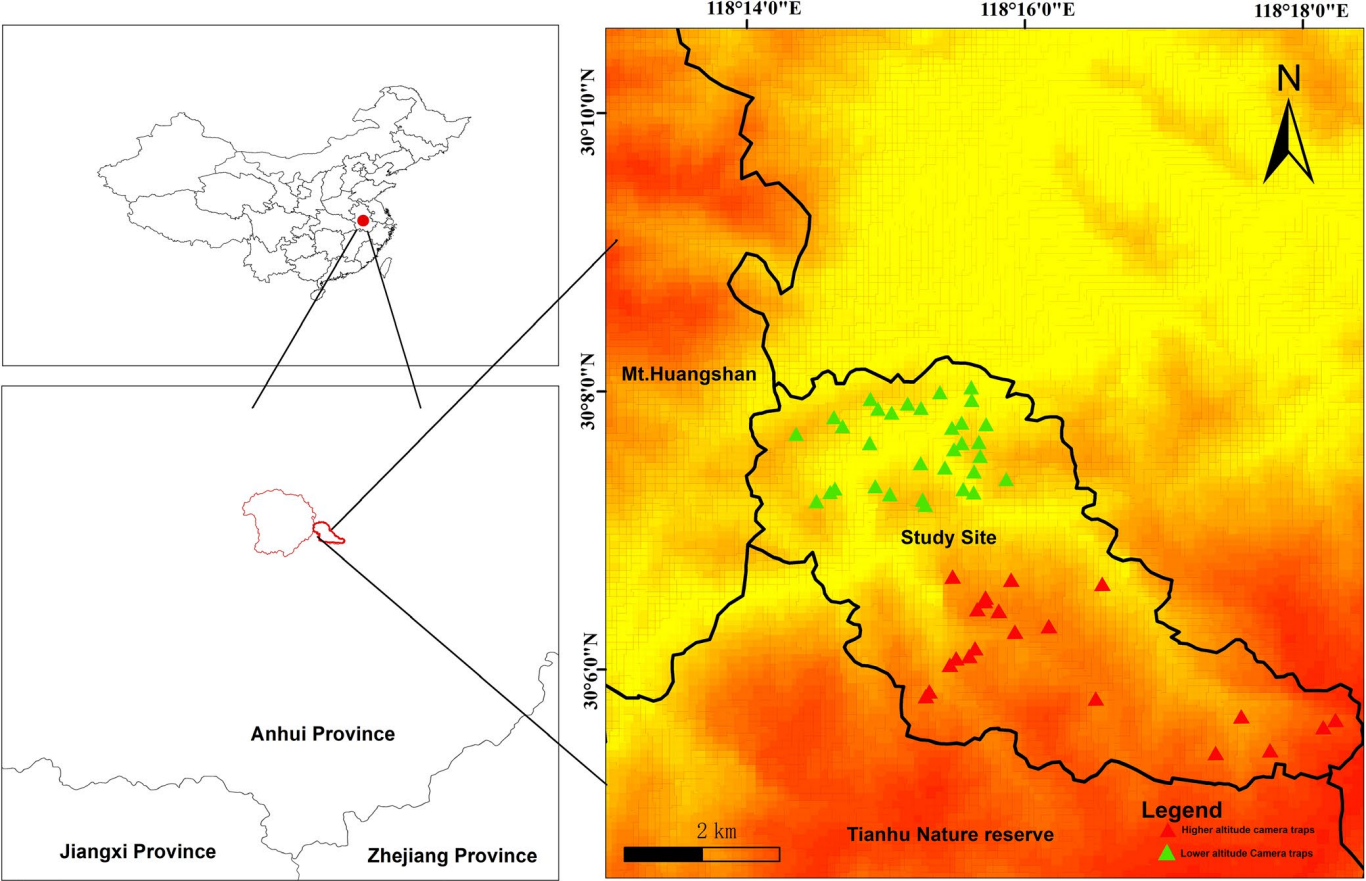
Study site and the distribution of camera traps in this study. The study site was situated at the boundaries of the Huangshan Mountains and Tianhu Nature Reserve. Triangles represent the effective monitoring points of infrared cameras. The higher altitude camera traps are marked as red triangles (total 21 traps). The lower altitude camera traps are marked as green triangles (total 31 traps, one camera was lost and replaced in another place near this camera traps)

We conducted our study in the Niejiashan Research Base (NRB) at Mt. Huangshan, Anhui Province, east‐central China (30°12′N, 118°27′E, 250–650 m above sea level), founded by the International Collaborative Research Center for Huangshan Biodiversity and Tibetan Macaque Behavioral Ecology, Anhui University, in 2017 (Figure 1). The aims of this study were to monitor the biodiversity and Tibetan Macaque behavioral ecology in Mt. Huangshan, including its surrounding areas. The NRB is located adjacent to Mt. Huangshan and Tianhu Nature Reserve (a provincial nature reserve in Anhui Province) and has a total area of 35.12 km^2^. In 1990, this place was designated a UNESCO World Heritage Site for being a site of scenic natural beauty. Huangshan was then declared a national park by the government and is now a major developed tourist destination in China. It is an important area in the pilot area of the great Mt. Huangshan National Park. It is also an important member of the 32 inland biodiversity conservation priority areas (Mt. Huangshan–Huaiyu Mountain) in China. The NRB is surrounded by mountains with steep slopes, with the altitude increasing from the northwest to the southeast. The intermontane plain is located in the lowland. Tianhu Mountain (1,217 m) is the main peak in the area. Due to inconvenience in transportation, the area is sparsely populated by humans. A large and intact subtropical evergreen deciduous broad‐leaved mixed forest survived. We predicted that habitat characteristics should be considered first and increase the size of protected areas second in nonprotected areas’ biodiversity conservation strategies as these areas are the last refuge for mammals can be found in the whole Mt. Huangshan–Huaiyu mountain area.

**FIGURE 2:**
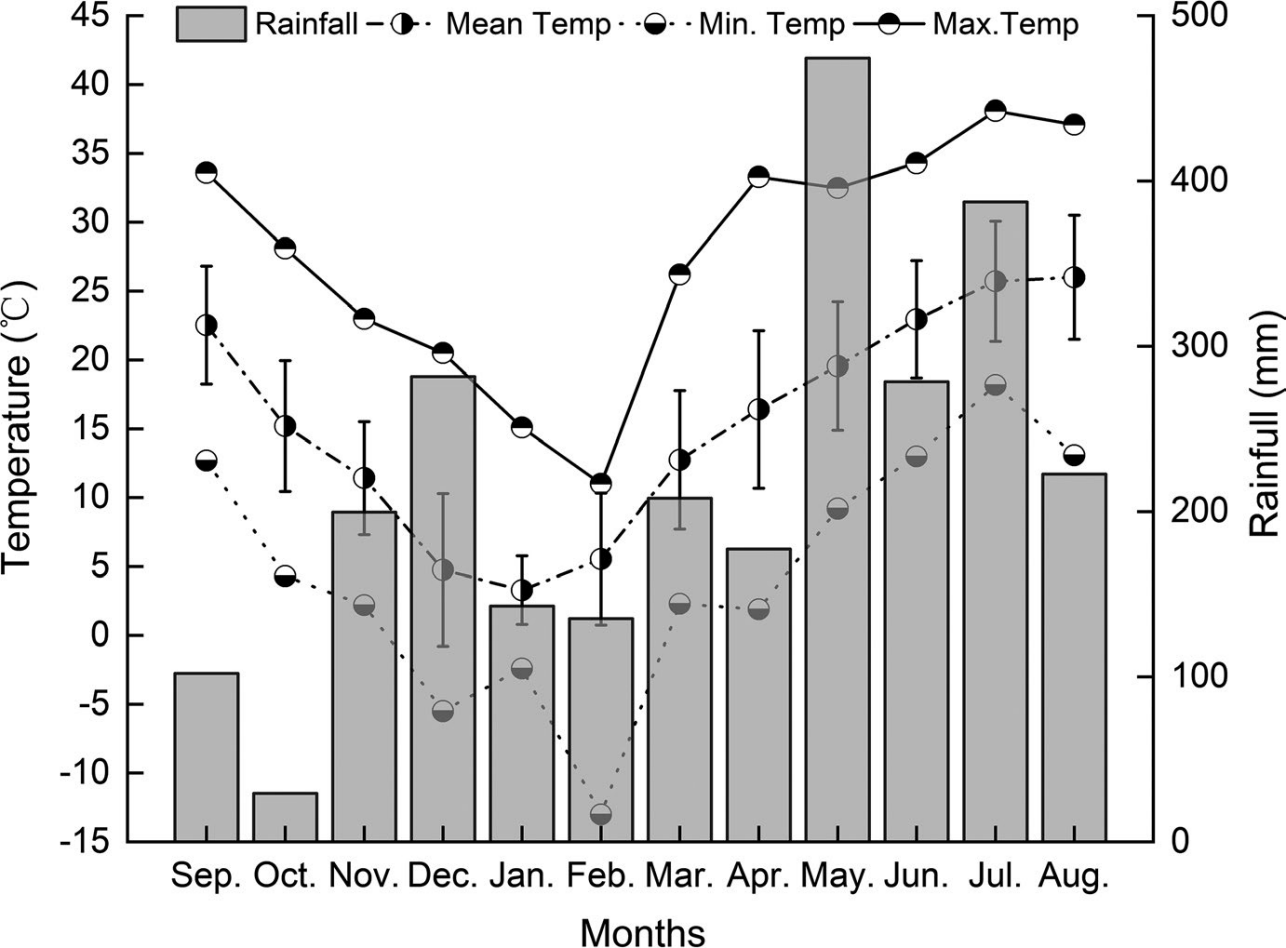
Monthly rainfall and maximum, minimum, and average temperature at Niejiashan Research Base during the study period

This nonprotected area is situated in a subtropical monsoon climate zone. The rainfall during September 2018 to August 2019 was 2,639.4 mm, the mean monthly rainfall during this period was 29.6–474.4 mm, and the mean temperature was 15.5°C with the highest temperature in July (38.1°C) and the lowest in February (−13.1°C) (Figure 2) (data acquired from the automatic weather station (QS‐3000) of NRB).

### 2.2 Data collection

#### 2.2.1 Camera traps

We first conducted the study in the higher altitude range (750–1,100 m) using six camera traps from June 2017 to June 2018. We then added 10 additional camera traps in nearby areas in July–October 2018. These cameras are mainly deployed to obtain a wider survey area and more recorded species; therefore, habitat environmental factors were not recorded at these camera trap locations (higher altitude camera traps are marked as red triangles in Figure 1, total 21 traps). From October 2018 to October 2019, we had to stop to check the camera traps in higher altitude areas and turned our monitoring efforts to lowland forest areas where 30 camera traps had been set up in total 31 traps (one camera was lost and replaced in another place near this camera traps in Table 1 and Figure 1, the green triangles), considering personnel security, government restraint, and laborious working environment. Therefore, a total of 36 infrared cameras (EREAGLE TRAIL CAMERA POWER: DC ~6–17 V; LENS: 7.45 mm E1) were used to cover 52 camera trap locations, for camera traps in the high‐altitude areas (total 21 traps), mainly takes into account the animal trails, human accessibility, vegetation type, elevation, topography, and other factors to detect as many animals as possible, for camera traps in the low‐altitude areas (total 31 traps) we considered more on microtopography, along various animal trails, and the interval between each camera was not <500 m (Figure 1). It should be noted that each camera trap location was only equipped with one camera, fixed on a thick tree, 50–60 cm high, with a belt. The higher place of cameras on the tree may have reduced the capture rates of smaller species but likely did not affect our ability to find for the medium‐sized and large‐sized terrestrial or semiterrestrial mammals (Granados et al., 2016; Widness & Aronsen, 2018). The cameras were set to take three pictures and a 10‐s video at intervals of 0.05 s. Continuous camera monitoring work started from June 2017 to October 2019, and each effective camera trap location was active for at least 30 days. We also pointed cameras in a southerly direction to avoid direct sunlight (Sukma et al., 2019). The same sampling points were used every month. We checked the camera every month to confirm that it was still working (it could have been damaged by some large animal, such as primates). The working time of each camera was calculated according to their actual working time, that is, excluding the time of failure due to issues with the battery or other such factors.

**TABLE 1:** Characteristics of camera traps. Date refers to the time when the cameras started working. Altitude and Area refer to the altitude and area covered by the cameras, respectively. Trap days represent the total active time of the camera traps; species is the total number of species photographed by the cameras

Date	Camera traps	Altitude (m)	Area (km^2^)	Trap days	Species
2017/06–2018/06	6	724–1,100	4.12	1,231	12
2018/07–2018/10	16	449–1,020	10.8	3,467	13
2018/10–2019/10	30	250–780	11.15	4,787	18

#### 2.2.2 Habitat characteristics

We used stratified random sampling to divide forests and habitats into three different types or levels in the low‐altitude areas. From October 2018 to October 2019, a total of 30 plots (20 m × 20 m each; the total area of 11.15 km^2^) were set up according to the camera location in the low‐altitude areas. Among these, 10 plots were in evergreen broad‐leaved forests, eight plots were in deciduous broad‐leaved forests, and 12 plots were in mixed forests (it also takes into account altitude, topography, and human accessibility). Since we used 30 infrared cameras to conduct a comprehensive periodic survey of the low‐altitude area from October 2018 to October 2019, we investigated the habitat characteristics at the 30 camera traps in the low‐altitude areas, and the data of high‐altitude areas were used only for species counts (Table 2). These factors include hidden conditions, water sources, and food sources related to the presence or abundance of mammals in our study area and elsewhere (Badgley, 2010; Liu et al., 2017; Sukma et al., 2019). Altitude, slope, aspect, and slope position, which were measured on each site, can influence the occurrence and relative abundance of the medium‐sized and large‐sized terrestrial or semiterrestrial mammals (Bogoni et al., 2016; Claridge & Barry, 2000). Various characteristics of the forest vegetation (such as forest type, DBH, tree canopy, tree density, tree height, shrub coverage, shrub height, and herb coverage) important in influencing the distribution and abundance of mammals were also measured (Bennett, 1993; Claridge & Barry, 2000). The intensity of human use/activities, distance to roads, and human habitation were not measured on account of the forested area, which is defined as an area with the least or zero number of human activities and highest distance to human settlement.

**TABLE 2:** Definition and category of habitat characteristics in 30 camera trap locations in the low‐altitude areas

Habitat characteristics	Definition	Category
Altitude	Location of camera traps	–
Aspect of slopes	Via an electronic compass E = east = 45°–135°; S = south = 135°–225°; W = west = 225°–315°; *N* = north = 315°–360° and 0°–45°	E, S, W, *N*
Slope position	Different parts of the mountains	Upper, middle, lower positions
Slope gradient	Gentle slope (≤30°); slight slope (30°–60°); steep slope (≥60°)	Gentle slope, slight slope, steep slope
Distance from water source	Near (≤50 m), mid‐distance (50–100 m), and far (≥100 m)	Near, mid‐distance, far
Forest types	Evergreen broad‐leaved forests; deciduous broad‐leaved forests; mixed forests	–
DBH	diameter at breast height	≤15 cm, ≥30 cm, 15–30 cm
Tree canopy	Degree of coverage of tree crown	≤25%, 25%–50%, 50%–75%, ≥75%
Tree density	Number of all trees with DBH ≥5 cm	–
Tree height	Actual height of the tree as perceived	–
Shrub coverage	Coverage degree of shrub crown	≤5%, ∼5%–10%, ∼10%–15%
Shrub height	Actual height of the tree as perceived	0–1 m, 2–3 m, 3–4 m, 5–6 m, ≥7 m
Herb coverage	Coverage degree of herb crown	≤2%, ∼2%–4%, and ∼4%–5%

### 2.3 Data analysis

After field survey data were collected, species identification was conducted on the photographs and videos using China's mammal diversity (second edition) to refer to the classification system of mammalian species (Jiang et al., 2017). Species of the IUCN Red List assessment level were identified (IUCN, 2017). For each effective monitoring site, all photographs and videos at intervals of 30 min (for small‐sized mammals) or 60 min (for larger‐sized mammals, as they have strong mobility) were combined as a valid statistic (effective detection or independent photographs) for the species. The relative abundance index (RAI) was calculated based on the effective detection of each species (Burton et al., 2015; O’Connell et al., 2011). Individual species RAI was calculated as follows: RAI = (Independent photographs/total number of trap days) × 100 (Blake & Loiselle, 2018). We used the species accumulation curve to assess sampling effort (Colwell & Elsensohn, 2014). Species richness gradually stabilized with increasing numbers of traps and traps day, nearing 18 species.

A chi‐square goodness‐of‐fit test was used to test the relationship between habitat characteristics and our measures of the RAI of different mammal species (including 11 species, with more than 10 individual photographs of each species). Linear model was used to create the global model, including camera trap location habitat characteristics (altitude, aspect of slope, slope position, slope gradient, distance from water source, forest type, diameter at breast height (DBH), tree canopy, tree density, tree height, shrub coverage, shrub height, and herb coverage; Table 2). We compared support for a total of 49 models of mammal species RAI, including a null (intercept‐only) model, for all analyses because the information‐theoretic framework (an information criterion corrected for small sample size) makes up for many defects in the use of conventional stepwise regression analysis. Based on the AIC determination method, model selection and multimodel inference were used to explore the determinants of the diversity and composition of mammals (Burnham & Anderson, 2002; Palmer & Koprowski, 2015). Before logistic regression analyses, independence tests were conducted using a nonparametric Spearman rank correlation of habitat characteristic data. All factors related to habitat characteristics (*N* = 13) were selected into the model during the model construction of each species. The function *glmulti* in the “glmulti” package was used to screen all possible models and select the optimal model. If Δ AICc > 2, then the end model was chosen, namely the optimal model for the first model; for all the models, *MuMIn* in *lm* average function model was used to list all the possible models. Analyses were carried out in R for windows version 3.3.0 (R Core Team, 2016). The significance level was set at *p* = 0.05.

## 3 RESULTS

### 3.1 Species composition and diversity

We collected a total of 2,212 independent photographs of mammal species, representing a total of 18 species, over 9,485 trap days. According to the IUCN Red List of Species, there were three near‐endangered species (Tibetan macaque (*Macaca* *thibetana*) [RAI = 2.58], Hog‐badger (*Arctonyx collaris*) [RAI = 1.14], and Serow (*Capricornis sumatraensis*) [RAI = 0.05]) and one endangered species (Black muntjac (*Muntiacus crinifrons*) [RAI = 0.03]). Together, they accounted for 22.2% of the total number of species encountered. Four species (Edwards's long‐tailed giant rat (*Leopoldamys edwardsi*) [RAI = 10.97], Reeve's muntjac (*M*. *reevesi*) [RAI = 8.04], *Tibetan macaque*, and Rhesus monkey (*M*. *mulatta*)) showed higher abundance–activity indices (scoring over 2%) and were deemed dominant. The RAI for White‐bellied rat (*Niviventer niviventer*), *Hog‐badger*, Chinese ferret‐badger (*Melogale moschata*), Wild pig (*Sus scrofa*), Masked palm civet (*Paguma larvata*), Red‐bellied tree squirrel (*Callosciurus erythraeus*), and Maritime striped squirrel (*Tamiops maritimus*) accounted for more than 0.1 each, and thus, they were considered to be common species. Siberian weasels (*Mustela sibirica*), Serow, Yellow‐throated marten (*Martes flavigula*), Chinese hares (*Lepus sinensis*), Black muntjac, European hedgehog (*Erinaceus europaeus*), and European badger (*Meles meles*) each accounted for <0.1, and as such, they were considered to be rare species (Table 3). The camera traps differed in the RAI of species (chi‐square goodness‐of‐fit test: *df* = 10, χ^2^ = 119.77, *p* = 0.000).

**TABLE 3:** Composition, IUCN conservation status, and relative abundance index (RAI) of mammal species in nonprotected areas during June 2017–October 2019; the photographs were the total number of independent records (2,212); trap days were given by all the normal capture days (9,485)

Mammals	IUCN (2017)	RAI
Primates		
Cercopithecidae		
*Macaca thibetana*	NT	2.58
*Macaca mulatta*	LC	2.38
Carnivora		
Mustelidae		
*Martes flavigula*	LC	0.04
*Mustela sibirica*	LC	0.07
*Melogale moschata*	LC	0.94
*Meles leucurus*	LC	0.01
*Arctonyx collaris*	NT	1.14
Viverridae		
*Paguma larvata*	LC	0.61
Artiodactyla		
Suidae		
*Sus scrofa*	LC	0.77
Cervidae		
*Muntiacus reevesi*	LC	8.04
*Muntiacus crinifrons*	VU	0.03
Bovidae		
*Capricornis sumatraensis*	NT	0.05
Lagomorpha		
Leporidae		
*Lepus sinensis*	LC	0.04
Rodentia		
Muridae		
*Leopoldamys edwardsi*		10.97
*Niviventer niviventer*		1.30
Sciuridae		
*Tamiops maritimus*	LC	0.25
*Callosciurus erythraeus*	LC	0.31
Insectivora		
Erinaceidae		
*Erinaceus europaeus*		0.03

### 3.2 Habitat characteristic requirements of different mammals

We characterized the habitat of where mammals were found at 30 camera trap sites with 13 habitat characteristics. Univariate analyses revealed that the trap sites were significantly different in terms of 13 habitat characteristics (*p* = 0.000). There was significant correlation between species richness (the total number of species) and altitude (Rs = 0.628, *p* = 0.000), slope position (Rs = −554, *p* = 0.000), distance from water source (Rs = 0.162, *p* = 0.015), tree density (Rs = 0.338, *p* = 0.000), tree coverage (Rs = −0.504, *p* = 0.000), DBH (Rs = −0.318, *p* = 0.000), tree height (Rs = −0.278, *p* = 0.000), shrub coverage (Rs = −0.442, *p* = 0.000), and shrub height (Rs = −0.159, *p* = 0.016) in different camera trap sites.

We ran linear models with the RAI and habitat characteristic factors (*N* = 13) of 11 mammals. The model showed that each mammal had different habitat characteristics (Table 4), and each species showed a significant correlation with its own habitat characteristics (Table 5). Species that were involved in topographic features (altitude, slope, slope position, slope gradient, distance from water sources) were Tibetan macaques (*M*. *thibetana*) (slope gradient: β ± *SE* = 2.00 ± 0.94, *t* = 2.13, *p* = 0.04), which preferred steep hills; Rhesus monkey (*M. mulatta*) (altitude: β ± *SE* = 0.02 ± 0.01, *t* = 2.51, *p* = 0.02), which preferred higher mountains; Reeve's muntjac (*M*. *reevesi*) (altitude: β ± *SE* = 0.03 ± 0.02, *t* = −2.20, *p* = 0.04; slope: β ± *SE* = −0.35 ± 1.37, *t* = −2.81, *p* = 0.01; slope gradient: β ± *SE* = −7.92 ± 1.52, *t* = −5.22, *p* < 0.001), which preferred higher mountains with gentle sunny slopes; Maritime striped squirrel (*T. maritimus*) (slope position: β ± *SE* = −0.37 ± 0.12, *t* = −3.04, *p* = 0.005; water: β ± *SE* = −0.24 ± 0.10, *t* = −2.461, *p* = 0.02), which preferred lower slopes with long distance water sources; Wild pig (*S. scrofa*) (slope position: β ± *SE* = −0.52 ± 0.20, *t* = −2.68, *p* = 0.01), which preferred lower slopes; Hog‐badger (*A. collaris*) (slope position: β ± *SE* = −0.67 ± 0.33, *t* = −2.01, *p* = 0.06), which preferred lower slopes; and Edwards's long‐tailed giant rat (*L. edwardsi*) (slope gradient: β ± *SE* = −4.85 ± 2.73, *t* = −1.78, *p* = 0.06), which preferred gentle hills. Species that were involved in forest features (forest types, tree canopy, density, height, DBH, shrub height and coverage, herb coverage) were *Rhesus monkey* (forest types: β ± *SE* = 1.24 ± 0.56, *t* = 2.21, *p* =0.036), which preferred deciduous broad‐leaved and evergreen forests; White‐bellied rat (*N. niviventer*) (shrub coverage: β ± *SE* = −1.52 ± 0.39, *t* = 3.94, *p* < 0.001), which preferred a lower degree of shrub coverage, usually ≤5%; Wild pig (tree density: β ± *SE* = 0.05 ± 0.001, *t* = 5.68, *p* < 0.001; tree canopy: β ± *SE* = −0.82 ± 0.28, *t* = −2.88, *p* < 0.001; shrub coverage: β ± *SE* = 0.58 ± 0.16, *t* = 3.70, *p* < 0.001), which preferred many trees with lower canopy and higher shrub coverage; Reeve's muntjac (tree density: β ± *SE* = 0.40 ± 0.08, *t* = 5.05, *p* < 0.001; shrub height: β ± *SE* = −3.52 ± 1.46, *t* = −2.41, *p* = 0.02; herb coverage: β ± *SE* = 3.08 ± 1.18, *t* = 2.62, *p* = 0.02), which preferred many trees with lower shrub height and higher herb coverage; and Masked palm civet (*P. larvata*) (forest types: β ± *SE* = 0.39 ± 0.23, *t* = 1.70, *p* < 0.10; shrub height: β ± *SE* = −0.66 ± 0.26, *t* = −2.60, *p* = 0.02), which preferred deciduous broad‐leaved forests with lower shrub height. Chinese ferret‐badger (*M*. *moschata*) (tree canopy: β ± *SE* = −1.29 ± 0.65, *t* = −1.98, *p* = 0.06; DBH: β ± *SE* = 0.20 ± 0.12, *t* = 1.68, *p* = 0.11) and Hog‐badger (tree density: β ± *SE* = 0.04 ± 0.02, *t* = 2.22, *p* = 0.04; herb coverage: β ± *SE* = 1.10 ± 0.25, *t* = 4.37, *p* < 0.001) preferred many trees with higher herb coverage, and Red‐bellied tree squirrel (*C*. *erythraeus*) (tree density: β ± *SE* = 0.02 ± 0.01, *t* = 2.05, *p* = 0.05; tree canopy: β ± *SE* = −1.67 ± 0.26, *t* = −4.53, *p* < 0.001; shrub coverage: β ± *SE* = −0.20 ± 0.12, *t* = 1.7, *p* = 0.10) preferred many trees with lower canopy and shrub coverage (Table 5).

**TABLE 4:** Model selection and measures for models using logistic regression to explain different needs of habitat characteristics between 11 different mammal species in nonprotected areas

Model	*K*	Log likelihood	AICc	ΔAICc	AICcWt	Wi
*Macaca thibetana*						
Slope + slope gradient	4	−82.25	174.11	0	0.32	0.32
Slope gradient	3	−83.69	174.3	0.19	0.29	0.61
Null	2	−84.98	174.41	0.3	0.27	0.88
Slope	3	−84.59	176.09	1.98	0.12	1
*Macaca mulatta*						
Altitude + forest types	4	−69.98	149.56	0	0.65	0.65
Altitude	3	−72.48	151.88	2.31	0.2	0.85
Forest types	3	−73.12	153.17	3.61	0.11	0.96
Null	2	−75.32	155.09	5.52	0.04	1
*Paguma larvata*						
Forest types + shrub height	4	−42.36	94.32	0	0.46	0.46
Shrub height	3	−43.89	94.7	0.38	0.38	0.84
Null	2	−46.43	97.31	2.99	0.1	0.94
Forest types	8	−45.7	98.32	4	0.06	1
*Melogale moschata*						
Tree canopy + DBH	4	−53.57	116.74	0	0.34	0.34
DBH	3	−55.05	117.02	0.29	0.3	0.64
Null	2	−56.73	117.91	1.17	0.19	0.83
Tree canopy	3	−55.6	118.12	1.38	0.17	1
*Arctonyx collaris*						
Tree density + slope position + herb coverage	5	−48.6	109.7	0	0.55	0.55
Tree density + herb coverage	4	−50.77	111.13	1.44	0.27	0.82
Tree density + slope position	4	−51.2	112.01	2.31	0.17	0.99
Tree density	3	−55.45	117.82	8.12	0.01	1
*Sus scrofa*						
Tree density + slope position + tree canopy + shrub coverage	6	−27.21	70.08	0	0.82	0.82
Tree density + tree canopy + shrub coverage	5	−31	74.5	4.42	0.09	0.91
Tree density + slope position + tree canopy	5	−31.52	75.45	5.46	0.05	0.96
Tree canopy + shrub coverage	4	−34.06	77.72	7.65	0.02	0.98
Tree density + tree canopy	4	−35.17	79.94	9.87	0.01	0.99
Slope position + tree canopy + shrub coverage	5	−33.76	80.02	9.94	0.01	1
*Muntiacus reevesi*						
Altitude + slope + slope gradient + tree density + shrub height + herb coverage	8	−91.31	205.47	0	0.42	0.42
Slope + slope gradient + tree density + shrub height + herb coverage	7	−94.17	207.43	1.96	0.16	0.58
Altitude + slope + tree density + shrub height + herb coverage	7	−94.69	208.46	2.99	0.09	0.67
Slope + tree density + shrub height + herb coverage	6	−96.86	209.38	3.9	0.06	0.73
Altitude + slope gradient + tree density + shrub height + herb coverage	7	−95.23	209.54	4.07	0.05	0.78
Altitude + slope + slope gradient + shrub height + herb coverage	7	−95.73	210.54	5.07	0.03	0.81
Slope + slope gradient + shrub height + herb coverage	6	−97.45	210.56	5.09	0.03	0.84
Slope gradient + tree density + shrub height + herb coverage	6	−97.63	210.92	5.45	0.03	0.87
Altitude + slope gradient + shrub height + herb coverage	6	−97.69	211.03	5.56	0.03	0.9
Slope gradient + shrub height + herb coverage	5	−99.38	211.27	5.8	0.02	0.92
*Tamiops maritimus*						
Water + slope position	4	−15.72	41.03	0	0.74	0.74
Water	3	−18.75	44.43	3.39	0.14	0.88
Null	2	−20.45	45.34	4.31	0.09	0.97
Slope position	3	−20.12	47.16	6.13	0.03	1
*Callosciurus erythraeus*						
Tree density + tree canopy + shrub coverage	5	−25.13	62.75	0	0.42	0.42
Tree canopy + shrub coverage	4	−26.69	62.98	0.23	0.37	0.79
Tree density + shrub coverage	4	−27.38	64.36	1.61	0.19	0.98
Shrub coverage	3	−31.03	68.99	6.23	0.02	1
*Niviventer niviventer*						
Shrub coverage	3	−63.77	134.46	0	1	1
Null	2	−70.38	145.21	10.57	0	1
*Leopoldamys edwardsi*						
Slope gradient	3	−117.12	241.16	0	0.59	0.59
Null	2	−118.72	241.88	0.72	0.41	1

Notes

Models were ranked in order of increasing AICc values.

Abbreviations: AICc, Akaike's information criterion values; AICcWt, relative strength of support for each model; ΔAICc, difference between the specified model and the optimal model; *K*, number of parameters; W_i_, AICc model weight

**TABLE 5:** Results of the linear model examining whether the relative abundance index of 11 mammal species significantly predicted their habitat characteristic needs

Habitat characteristic	Estimate	*SE*	*t*	*p*
*Macaca thibetana*				
Intercept	−5.09	3.44	−1.48	0.15
Slope gradient	2	0.94	2.13	0.04*
Slope	1.47	0.89	1.64	0.11
*Macaca mulatta*				
Intercept	−7.05	2.69	−2.63	0.01*
Altitude	0.02	0.01	2.5	0.02*
Forest types	1.24	0.56	2.21	0.04*
*Paguma larvata*				
Intercept	2.18	0.94	2.31	0.03*
Forest types	0.39	0.22	1.7	0.1
Shrub height	−0.66	0.26	−2.6	0.02*
*Melogale moschata*				
Intercept	2.96	3.02	0.98	0.34
Tree canopy	−1.29	0.65	−1.98	0.06
DBH	0.2	0.119	1.675	0.11
*Arctonyx collaris*				
Intercept	−1.38	1.49	−0.92	0.37
Slope position	−0.67	0.33	−2.01	0.06
Tree density	0.04	0.02	2.22	0.04*
Herb coverage	1.1	0.25	4.37	0.000***
*Sus scrofa*				
Intercept	0.53	1.31	0.40	0.70
Tree density	0.046	0.01	5.68	0.000***
Slope position	−0.52	0.2	−2.68	0.01*
Tree canopy	−0.82	0.28	−2.88	0.008**
Shrub coverage	0.58	0.16	3.7	0.001**
*Muntiacus reevesi*				
Intercept	34.5	10.59	3.26	0.003**
Altitude	−0.03	0.02	−2.2	0.04*
Slope	−3.85	1.37	−2.81	0.01*
Slope gradient	−7.92	1.52	−5.22	0.000***
Tree density	0.4	0.08	5.05	0.000***
Shrub height	−3.52	1.46	−2.41	0.02*
Herb coverage	3.08	1.18	2.62	0.02*
*Tamiops maritimus*				
Intercept	1.35	0.36	3.8	0.001***
Water	−0.24	0.1	−2.46	0.02*
Slope position	−0.37	0.12	−3.04	0.005**
*Callosciurus erythraeus*				
Intercept	4.53	1.20	3.77	0.000***
Tree density	0.02	0.01	2.05	0.05*
Tree canopy	−1.17	0.26	−4.53	0.000***
Shrub coverage	−0.2	0.12	−1.69	0.1
*Niviventer niviventer*				
Intercept	5.82	1.17	4.96	0.000***
Shrub coverage	−1.52	0.39	−3.94	0.000***
*Leopoldamys edwardsi*				
Intercept	22.13	6.77	3.27	0.002**
Slope gradient	−4.85	2.73	−1.78	0.09

Notes

Significant differences: *0.001<*p* < 0.005,**p < 0.001,***p = 0.000.

## 4 DISCUSSION

### 4.1 Composition and diversity in nonprotected areas

Our data showed that more mammals were present in the nonprotected area studied; we found 18 mammal species captured by 36 cameras in a span of two years, which were more than the number of species reported in Mt. Huangshan (14 species) (Liu et al., 2017), Anhui Jiulongfeng Provincial Nature Reserve (10 species) (Wang et al., 2015), Anhui Guniujiang National Nature Reserve (12 species) (Fang, 2017), and Anhui Qingliangfeng National Nature Reserve (nine species) (Li et al., 2017). Furthermore, we also found three near‐endangered species (Tibetan macaque (*M. thibetana*), Hog‐badger (*A. collaris*), and Serow (*C. sumatraensis*)) and one endangered species (Black muntjac (*M. crinifrons*)), which according to the IUCN Red List of Species are considered to be live in the protected areas. Although the study site size, the number of infrared cameras, and duration of monitoring had some influence on mammal diversity, the area (35.12 km^2^) was monitored for 2 years, revealing that this nonprotected area was inhabited by more mammal species than protected areas.

### 4.2 The disappearance of "empty forests" phenomenon

In our study area, with the implementation of the national policy of returning grain plots to forestry, habitat destruction caused by destructive grazing and logging has been gradually replaced by ecotourism, resulting in forest restoration over time. More mammal species live in here also confirmed the disappearance of the "empty forests" phenomenon, such as the highest rates recorded for Edwards's long‐tailed giant rat (*L. edwardsi*), White‐bellied rat (*N. niviventer*), Reeve's muntjac (*Muntiacus reevesi*), and Wild pigs (Andresen et al., 2018; Barrios‐Garcia & Ballari, 2012; Bhattarai & Kindlmann, 2011; Cheyne et al., 2016; Vanthomme et al., 2013). These nonprotected areas also showed some characteristics of habitat degradation—for example, the absence of large carnivorous wildlife and decrease in the number of rare and endangered species. No large carnivorous animal (such as Black bear (*U*. *thibetanus*) and Clouded leopard (*N*. *nebulosa*)) was found in this study. They may have become extinct in this area during the 1980s due to excessive hunting, abuse of rodenticide, and habitat degradation (Wang, 1990). Even the small Leopard cat (*Prionailurus bengalensis*) that was recently found in Mt. Huangshan (Liu et al., 2017) may have become extinct here. Predatory mammals can result in detrimental effects on the survival of other species. Loss of such predators can then have a variety of catastrophic effects (Chiang et al., 2014; Kolchin, 2018; Watanabe & Izawa, 2005). Therefore, these restored forests require immediate conservation and management intervention, to increase the territory size of the endangered specialist species and ultimately increase biodiversity.

### 4.3 Habitat characteristics for different species

Furthermore, models showed that each mammal preferred different habitat characteristics (Table 4), and each species showed a significant correlation with its own habitat characteristics (Table 5). However, we also found some unexpected results: There was no strong habitat dependency of these species, and no single species was associated with all the characteristics. At the most, there were only six habitat‐related factors (such as for Reeve's muntjac), and most animal species were related to 1–3 habitat characteristics. These habitat characteristics—mostly natural habitat properties including altitude, slope, slope position, and slope gradient—are thought to be hard to destroy (Badgley, 2010; Qian et al., 2009). However, there were some species, such as Wild boar, that were associated with forest habitat characteristics (tree, shrub, herb) and preferred to live in forests with higher tree density, lower tree canopy, and higher shrub coverage. The vulnerability of forests and their importance to animals have been demonstrated in many studies (Bai et al., 2020; Barr & Biernat, 2020; Blake & Loiselle, 2018; McShea et al., 2009; Tédonzong et al., 2019), providing more evidence for the establishment of protected areas (increase in the size of protected areas). In this study, we found that increasing the size of protected areas may effectively protect species such as Reeve's muntjac and Wild boar (specialist species), which were highly dependent on habitat characteristics. Moreover, some species such as Tibetan macaque and Rodents (generalist species) have a high adaptability to different kinds of habitat, including anthropogenic areas (Klass et al., 2020; Mekonen, 2020). In some studies, it has been shown that the most crucial factors affecting the population decline of these species are illegal trade and excessive capturing and slaughtering. In these cases, it is less important to focus on habitat characteristics for the conservation of these species.

## 5 CONCLUSIONS

The establishment of protected areas with an increase in protected area size is a challenge to ensure the long‐term survival of many native species. For protected area planners and managers, the key is to know which habitat characteristics are important for the species occurring within that area and being able to make better decision regarding whether to increase territory size or increase other protective measures for pertinent species. Our study highlights the importance of habitat characteristics in the establishment of new protected areas for animals in nonprotected habitats. Without a specific focus on the habitat characteristics of different species, the establishment of protected areas may potentially be unsuccessful or may incur an exceedingly high cost.

Specifically, the importance of habitat characteristics of other species cannot be neglected for the conservation of an important species. Because different species have different needs for habitat characteristics, conservation strategies that favor only key species may cause populations of other sympatric species to decline. Recognizing habitat characteristic requirements of different species will specifically be important for conservation of mammalian species.

According to the results of this study, in order to effectively protect the diversity of mammals in this area, protected area planners and managers should consider protecting species such as Masked palm civet (*P. larvata*), Chinese ferret‐badger (*M*. *moschata*), Hog‐badger (*A. collaris*), Wild boar (*S. scrofa*), and Reeve's muntjac (*M*. *reevesi*) that are associated with the vegetation characteristics of their habitats. Appropriately increasing protected areas would provide the last refuge for these species.

## CONFLICT OF INTEREST

We have no conflict of interest to declare.

## AUTHOR CONTRIBUTION

**WenBo Li:** Data curation (lead); Formal analysis (equal); Investigation (lead); Methodology (equal); Visualization (equal); Writing‐original draft (lead); Writing‐review & editing (lead). **Jinhua Li:** Conceptualization (lead); Resources (lead); Supervision (equal); Visualization (equal); Writing‐review & editing (equal). **Peipei Yang:** Investigation (supporting); Writing‐review & editing (supporting). **Bowen Li:** Investigation (supporting); Writing‐review & editing (supporting). **Chao Liu:** Investigation (supporting); Writing‐review & editing (supporting). **Lixing Sun:** Methodology (supporting); Writing‐original draft (supporting).

### OPEN RESEARCH BADGES

This article has earned an Open Data Badge for making publicly available the digitally‐shareable data necessary to reproduce the reported results. The data is available at https://doi.org/10.6084/m9.figshare.13271714.v1.

## Data Availability

All data are available in the open figshare repository, and the link to the data is https://doi.org/10.6084/m9.figshare.13271714.
